# Advanced Telemedicine Training and Clinical Outcomes in Type II Diabetes: A Pilot Study

**DOI:** 10.1089/tmr.2021.0039

**Published:** 2022-01-11

**Authors:** Colton B. Merrill, Jason M. Roe, Kevin D. Seely, Benjamin Brooks

**Affiliations:** Rocky Vista University, Department of Research and Scholarly Activity, College of Osteopathic Medicine, Ivins, Utah, USA.

**Keywords:** telemedicine, telehealth, type II diabetes, subspecialization

## Abstract

**Background:** COVID-19 caused a dramatic increase in the scope and utilization of telemedicine. However, the sustainability of the permanent integration of telemedicine in the management of chronic disease beyond the pandemic is still enigmatic. The purpose of this retrospective chart review was to analyze the effect of advanced training in telemedicine on clinical outcomes in type II diabetes mellitus (T2DM) in the United States.

**Methods:** A retrospective chart review was conducted in 104 deidentified patients with diabetes from 28 specialized telemedicine agency physicians who had received specialized telemedicine training. After establishing exclusion criteria, the charts of 59 T2DM patients were evaluated. Glycated hemoglobin (HbA1c) percentage and body mass index (BMI) were used as quantitative endpoints. Visit consistency, mediation data, and compliance data were also studied.

**Results:** The mean change in HbA1c for the 42 patients who met the inclusion criteria for evaluating HbA1c (*n* = 42) was −0.429%. The largest decrease in HbA1c was 5.4%, and the most significant increase was 3.9%. The mean change in BMI for the 16 patients who met the inclusion criteria for evaluating BMI (*n* = 16) was −2.175 kg/m^2^. The largest decrease in BMI was 9.5 kg/m^2^ and the largest increase was +0.7 kg/m^2^. The average number of visits for patients with a decrease in HbA1c was 3.45. The average number of visits for patients with an increase in HbA1c was 2.62.

**Conclusions:** Outcomes of telemedicine providers with training are comparable with the standard of care. Advanced telemedicine training and its effect on clinical outcomes in the management of chronic disease warrant further investigation. For telemedicine to become a mainstay in U.S. medicine, a standard of best practices should be evaluated and available for providers who wish to continue telehealth care delivery.

## Introduction

Telemedicine is defined as the use of electronic information and telecommunication technology to deliver health care, including direct patient care, health education, and population health management.^1^ Virtual health care and telemedicine platforms provide chronic disease patients with enhanced access to medical services compared with the pretechnological era.^2^

Since the emergence of COVID-19, many physician groups and hospital systems have rapidly adopted telemedicine as an alternative to in-office visits at a time of social distancing.^3^ Telemedicine has been widely recognized for decades as a valuable method of improving access to health care services that would otherwise be difficult to obtain, perhaps due to location (rural and remote) or other barriers (frailty, lack of transportation, or other physical or mental health conditions).^4^

Our traditional health care system is heavily reliant on in-person consultations, perhaps causing inequity for individuals who are unable to physically attend.^5^ Indeed, the rapid adoption of telemedicine has been a prominent result of the COVID-19 pandemic to minimize the interruption of essential clinical services. As we look to the future beyond the COVID-19 pandemic, it is necessary to establish the criteria for suitability and optimal telemedicine delivery. This article explores the idea of advanced telemedicine training and telemedicine as a primary practice focus through examining patient outcomes of providers who have undergone advanced training.

Patients and providers have reported high levels of satisfaction after utilizing telemedicine.^6^ While there are clear benefits to the telemedicine medium for patient care such as improved time and resource efficiency in insulin titrations and counseling of glucose logs, the inherent limitations of telemedicine such as lack of physical examination and communication gaps created through technology difficulties raise concerns for long-term sustainability as a medium for the management of chronic conditions. These limitations include a lack of ability to conduct a proper physical examination as well as communication gaps created through technological difficulties.^7^ In addition, patient/physician interactions in telemedicine, as in other modes of care, result in varying levels of medical accountability.^8^

Inherent difficulties, such as these, increase risk and necessitate continuing medical education curricula or even the establishing of a telemedicine subspecialty for physicians who intend to treat using telecommunication beyond the COVID-19 pandemic.

The national physician workforce is becoming increasingly stratified according to discipline both by formal credentials, such as specialty board certifications, and by circumstantial or preferential clinical practice emphasis.^9,10^ The requirements for establishing a subspecialty in internal medicine are based on many conditions, including (1) evidence that the new discipline has a definable body of knowledge, (2) a significant number of clinical training programs, and (3) a realistic expectation that clinical services in the subspecialty will improve patient care.

Traditionally, such certifications have required at least a year of authorized instruction.^9^ While the relationship between specialization or subspecialization and improved clinical outcomes is well established,^9^ this study aims to evaluate point number 3 concerning the discipline of telemedicine and tests the hypothesis that advanced training in telemedicine will improve patient care and clinical outcomes. Of importance to note, telemedicine is not currently an established subspecialty, nor does it have its own residency or fellowship training programs in the United States. However, there are physicians and physician groups who receive extra training in telemedicine and provide care by telemedicine exclusively, or for whom the significant majority of patient interaction occurs via telecommunication.

The use of telemedicine in the management of type II diabetes mellitus (T2DM) was chosen because it is well studied. Several studies have emphasized the benefits of telemedicine interventions for diabetes treatment.^11,12^ However, many of them use a variety of care modalities, such as teleconsultation and remote patient monitoring. A 2016 meta-analysis involving 55 randomized studies and over 9000 patients showed an A1c improvement in patients with T2DM who were older than 43 years and received teleconsultation.^11^ In a systematic review conducted in 2017, 111 randomized-controlled trials and almost 24,000 patients, telemedicine interventions were studied and showed modestly reduced glycated hemoglobin (HbA1c) compared with usual care.^12^

Patients with a higher baseline HbA1c, as well as trials that used text messaging or web portals for communication and programs that aided medication adjustment, had the greatest influence in meta-regression analysis. While the use of continuous glucose monitors for remote patient monitoring is becoming more common and is increasingly approved by insurance providers, the use of these technologies in conjunction with virtual care is yet to be critically evaluated.

To adhere to current American Diabetes Association standards, effective implementation of telemedicine in diabetes management would require successfully maintaining A1c levels below a 7% threshold.^13^ Additional limitations of these studies include whether the providers involved received special training in the use of telemedicine, or whether they were specialty-trained physicians in family medicine, internal medicine, endocrinology, or otherwise.

Understanding the known benefit of telemedicine in the management of T2DM, this study sought to evaluate not only telemedicine in the management of diabetes but the difference in outcomes of clinical management by a provider with advanced telemedicine training.

### Overview of T2DM management

The treatment goals for nonpregnant adult patients diagnosed with T2DM are to prevent or delay complications and maintain quality of life. This includes a patient-centered plan aimed at glycemic control and cardiovascular risk factor management.^14^ Intensive lifestyle modifications and patient education are initially warranted focused on weight reduction, diet, and exercise. Despite the clear benefit and results of these interventions, only a small percentage of patients can be treated on these alone.^15^ Therefore, HbA1c is accepted as a diagnostic test and as a test to evaluate the effects of treatment management.^16^

For most patients presenting with HbA1c at or above target level (i.e., >6.5% to 8%), oral pharmacologic therapy should be initiated at the time of T2DM diagnosis. Patients with severe and/or symptomatic hyperglycemia (HbA1c >9%), as well as those who fail initial monotherapy and have no other current complications, are suggested to have a second oral or injectable agent added, including a glucagon-like peptide 1 agonist and insulin as options.

**FIG. 1.**
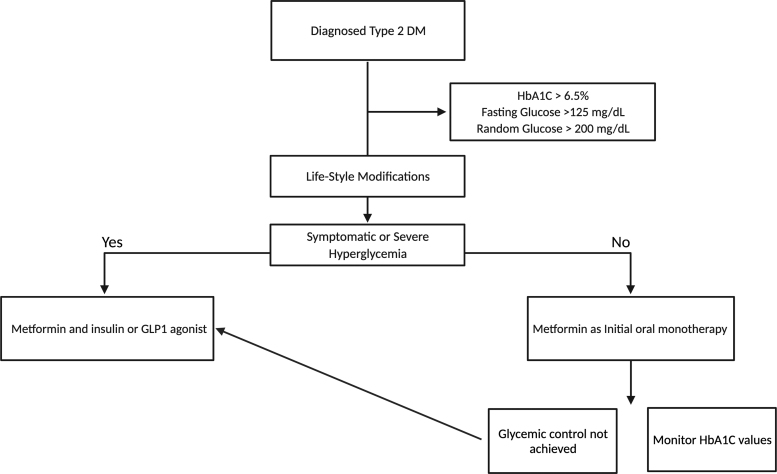
Therapeutic aims for nonpregnant adults with diagnosed T2DM. GLP-1, glucagon-like peptide 1; HbA1c, glycated hemoglobin; T2DM, type II diabetes mellitus.

It is common to obtain HbA1c values at least twice yearly in patients meeting glycemic goals and more frequently (quarterly) in patients whose therapy has changed or who are not meeting goals (Fig. 1). Effective management accepts shared decision-making, acknowledges individual fears, and is responsive to patient preferences and barriers. The shared decision-making is done by understanding the absolute benefit and risks associated with each treatment option.^13^

## Methods

### Design

This was a retrospective chart review. After querying a primary care physician group that sees patients exclusively via telemedicine, 104 deidentified patient data files were received, and a retrospective chart review was conducted. Data were synthesized, and findings from patient telemedicine visit records were assessed.

### Physicians

The 28 physicians included in this study are board-certified physicians in the United States, employed by a private telemedicine physician group, who have received additional training in telemedicine medicine designed and delivered by the group's chief medical officers, and who deliver health care primarily or exclusively via telecommunication. Providers included remote part-time telehealth physicians, remote full-time primary care/urgent care telehealth physicians, remote full-time nocturnist physicians, and remote nurse-practitioners.

### Patients

**FIG. 2.**
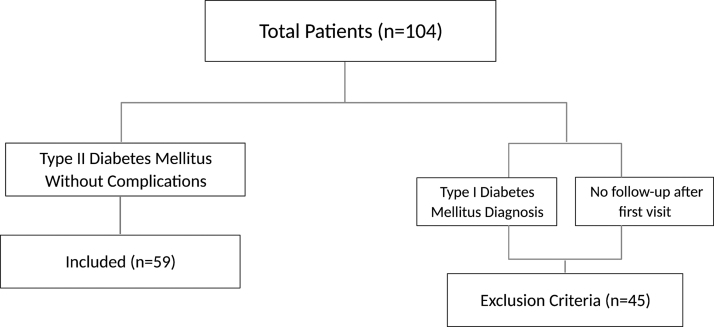
Flowchart of the initial patient inclusion and exclusion criteria.

The charts of 104 patients with diabetes were evaluated to determine whether the following criteria were met: (1) patients had an International Classification of Disease, Tenth Revision code of T2DM without complications and (2) patients met with a physician via telemedicine at least two times per year. Patients were excluded if they had one or more of the following: (1) type 1 diabetes diagnosis or (2) new patient visit with no subsequent follow-up visit (Fig. 2). Of note, these criteria are not only useful for inclusion and exclusion but are also intrinsically valuable data points.

**FIG. 3.**
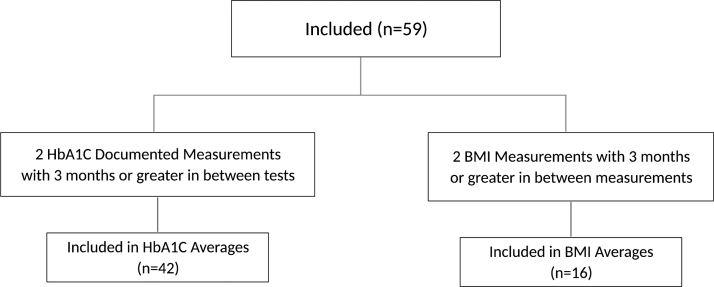
Flowchart of the final inclusion criteria for substudies of HbA1c and BMI. BMI, body mass index.

The final number of patients included in this study was 59. Patients were then subdivided based on having either (1) two HbA1c measurements with at least 3 months or greater in between tests or (2) two body mass index (BMI) measurements with 3 months or greater in between measurements (Fig. 3).

### Intervention

#### Physician training

Telemedicine training, in addition to experience and practice focus, included in-depth training on telemedicine and virtual care best practices, training on the telemedicine delivery platform, its logistics, and nuances. Physicians within the practice chose to take additional hours of training in telehealth-specific continuing medical education credits. Some clinicians received additional training with the functional medicine institute, which gives them a greater perspective on treating the patient with a holistic and/or integrative approach. Most physicians in the group were specialized in family medicine, internal medicine, emergency medicine, hematology, or have additional certifications.

Furthermore, physicians were equipped with detailed, practical, and medically appropriate clinical protocols and instructions on how to use them in the setting of telemedicine. This included supplemental instruction on health information privacy training and best practices in the setting of telemedicine. Technical support and troubleshooting of software were not expected of the providers and were resolved by information technology staff. All training and software were designed with the physician/patient relationship at the center to enable and facilitate a user-friendly virtual interaction.

Components of the additional training available for these providers pertained to the use of text and chat visits, video visits, phone visits, synchronous visits (meaning real-time conversation), asynchronous visits (meaning the patient sends a message and the provider answers when available and *vice versa*), urgent care, ongoing care, and short-term care.

#### Patient care delivery

Primary diabetes management care was delivered remotely for time periods ranging from 1 to 5 years. Patients were always connected with the same physician, in contrast to something like an on-demand doctor app in which a patient is paired with an available provider at random. Contact between physicians and patients was carried out completely through secure text, phone, and video chat. The practitioner analyzed the patient's medical and family histories during the first visit.

Then, as with any establishment of care, the provider discusses the patients' medical problems and concerns, reviews nutrition, exercise, sleep, work, and other critical aspects of the patients' health, and orders pertinent blood tests for the patients to acquire a detailed picture of their health. Laboratories and tests were completed at facilities local to the patient, and results were sent to the ordering physician and discussed with the patient. Patients were allowed to securely text their doctor at any time, and the physicians responded to the patients' text messages at various points throughout each day. Communication was via the same virtual platform for all patients and providers.

### Data collection and interpretation

All patient data were compiled and deidentified according to predetermined contract agreements before transfer for evaluation. Data from 104 individuals with diabetes were obtained, including patient age, encounter date, current BMI, BMI at last visit, appointment type, laboratory date, laboratory tests, laboratory values, and medication list. After data retrieval, key points for evaluation were identified as a change in HbA1c with at least 3 months in between, change in BMI with at least 3 months in between, number of visits per year, and patient adherence to national diabetes treatment guidelines. Patient age was the only demographic information able to be collected. The change in HbA1c results, change in BMI, and the respective averages of each were calculated.

## Results

**FIG. 4.**
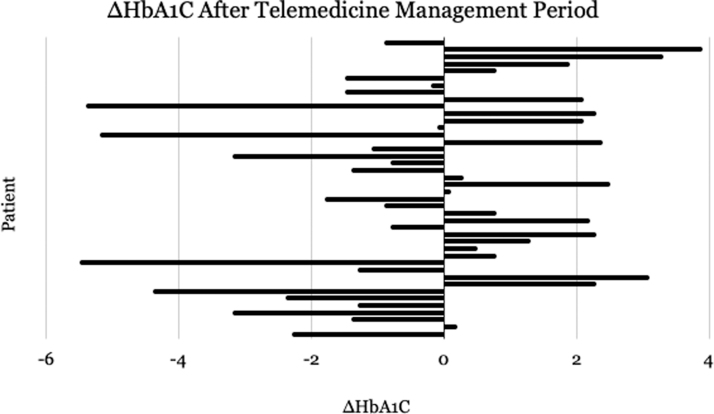
Change in glycated hemoglobin percentage over the duration of telemedicine delivery for 42 T2DM patients seen by physicians with advanced training in telemedicine best practices.

**FIG. 5.**
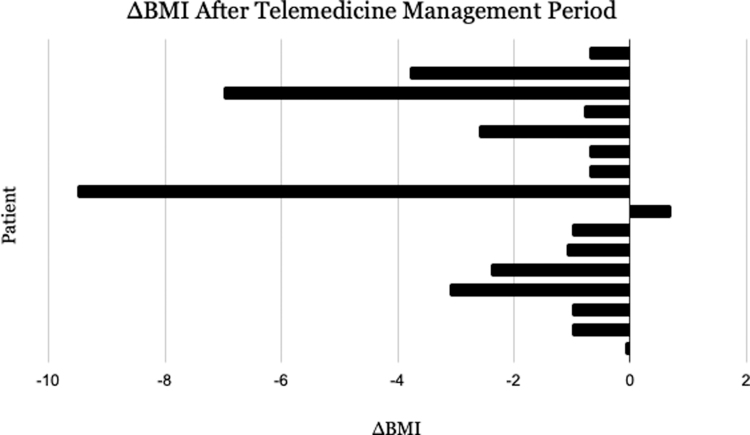
Change in BMI over the duration of telemedicine delivery for 16 T2DM patients seen by physicians with advanced training in telemedicine best practices.

The average age of the original 104 patients who were seen by telemedicine before exclusion was 48.8 years old. The mean change in HbA1c for the 42 patients who met the inclusion criteria for evaluating HbA1c (*n* = 42) was −0.429%. The largest decrease in HbA1c was 5.4%, and the most significant increase was 3.9% (Fig. 4). The mean change in BMI for the 16 patients who met the inclusion criteria for evaluating BMI (*n* = 16) was −2.175 kg/m^2^. The largest decrease in BMI was 9.5 kg/m^2^ and the largest increase was +0.7 kg/m^2^ (Fig. 5). The average number of visits for patients with a decrease in HbA1c was 3.45. The average number of visits for patients with an increase in HbA1c was 2.62.

Note that the duration of telemedicine delivery varied for each patient. This is an intentional deficit because each patient chose to continue or not to continue telemedicine visits at his or her will. Those with the largest decrease in HbA1c or BMI stayed with their telemedicine provider long enough to do so.

The patient who showed the largest decrease in BMI (9.5 kg/m^2^) also showed a HbA1c decrease of 1.1%. This patient had five documented formal telemedicine visits from 2019 to 2021. However, these visits do not include texts and other informal virtual communications between the doctor and physician. The patient who showed the largest increase in BMI (0.7 kg/m^2^) also showed an HbA1c decrease of 0.1%. This patient had three documented formal telemedicine visits in 2020, not including texts and other informal virtual communication.

The vast majority of patients with a documented change in BMI lost weight. As stated previously, only 16 of the 59 included T2DM patients had a documented change in BMI. The remaining 43 patient charts showed no net change in BMI, meaning their BMI remained stable throughout the duration of time in which they were seen by telemedicine or that their weight was not documented or presumed to be the same at every visit.

The patient who showed the largest decrease in HbA1c (5.4%) also showed a BMI decrease of 0.7 kg/m^2^, dropping from 28.1 to 27.4 kg/m^2^. This patient had six documented formal telemedicine visits from 2020 to 2021. As noted previously, these visits do not include texts and other informal virtual communications between the doctor and physician. The patient who showed the largest increase in HbA1c (3.9%) also showed a BMI decrease of 3.8 kg/m^2^. This patient had three documented formal telemedicine visits from 2017 to 2018, not including other informal virtual communications.

Twenty-two of the 43 patients showed a decrease in HbA1c, with the remaining half showing an increase in HbA1c. The average number of visits per year for the 22 patients who decreased their HbA1c measurement was 3.4 visits. Characteristics of patients who successfully decreased their HbA1c include regular follow-up with their physician, adherence to medications, regular exercise, and monitoring their nutrition.

## Discussion

Data analysis reveals several characteristics that predicted positive and negative clinical outcomes for T2DM patients being treated via a telemedicine medium. Of the 42 T2DM patients who met the inclusion criteria of at least two HbA1c measurements at least 3 months apart, 22 experienced an overall decrease in HbA1c values. Of note, these 20 patients met with their trained telemedicine physician virtually an average of 3.4 times. The 20 patients who showed an increase in HbA1c values had an average of 2.62 visits with their telemedicine physician. Therefore, a relationship is apparent between the number of patient visits and HbA1c management of T2DM via a telemedicine medium.

In contrast to these telehealth findings, one 2014 study showed that comparable improvements in A1c values correlated with the frequency of in-office visits. In the study, patients with diabetes who demonstrated the greatest decrease in A1c values met with their in-office physician for more than 6 consecutive visits with a visit gap of <1 month. Further analysis revealed that the average consecutive in-office visit lowered A1c levels by 0.25%, and the mean number of visits needed to achieve A1c <7% was eight.^17^ It is logical then to expect that frequent visits would add an increased load on physicians and health care resources.

Current guidelines provide little information on how frequently T2DM patients need to be seen by their health care providers, other than the current recommendation of A1c measurements being obtained every 3 months.^16^ Therefore, we believe it is reasonable to conclude that implementation of a telemedicine-based approach to diabetes care would ease this load on in-office physicians while simultaneously providing effective care options.

One interesting extrapolation was that of the 92 new patient visits for T2DM management, only 59 returned for a subsequent follow-up appointment. Due to a lack of postvisit patient survey data, conclusions regarding patient motivation for not returning for subsequent appointments are unclear. Analysis reveals that of the 33 patients who did not follow up, all received medication prescriptions at their initial appointment. Thus, a potential reason for the lack of follow-up could be the desire for a single time-efficient visit to obtain a prescription refill without having to travel and wait to see an in-office physician.

Of additional note, these 33 new patient visits occurred during the height of the COVID-19 pandemic. Therefore, a desire to minimize the risk of potential exposure to infected individuals at an in-person appointment is one of many potential incentives for seeking out a one-time telemedicine visit for a simple prescription refill. Data analysis also indicates that of these 33 patients, 6 had initial HbA1c values reported as less than the 5.7% recommended threshold. While these six patients had a history of T2DM and received prescriptions for diabetes medications from specialized telemedicine physicians, some of these patients may have neglected to return for a follow-up visit due to misguided justification that their HbA1c levels were normal and required no further follow-up.

Additional speculation could be that these six patients were in the process of finding a new physician, and a single telemedicine visit would fill their prescription needs sufficiently long enough to find a new in-office physician.

After data analysis, a current detriment to the development of telemedicine as an equal standard of care compared with the traditional in-office setting was established. The 33 patients who met with telemedicine specialists only one time and never returned for follow-up now have laboratory values and medications in their medical histories that will not be documented in their continuing record of medical care. It stands to reason then that their established in-office physicians likely have no knowledge of these patient interactions or the treatments and laboratories they received. These discrepancies in their medical records may appear insignificant for a single HbA1c value but could have drastic effects on a variety of medical conditions over time.

For telemedicine to advance to become an equally effective standard of care, a focus needs to be made to improve the transparency and communication of patient data and medical records between telemedicine providers and traditional in-office medical groups.

The major limitation of this study was the lack of a direct comparison group such as an in-office-based comparison group. This study could have been strengthened by adding a patient survey component; however, because of the retrospective nature of this study, a survey was not performed. Only one set of physicians and their patients was analyzed. Where there are multiple physician groups such as the one evaluated in the present study, a large multicenter study should be conducted after a large group of providers receive the same advanced telemedicine training. Another source of limitation was the lack of information about medication refills and adherence.

The scope of utility in varying patient populations and disease states needs to be further investigated. Furthermore, to realize its potential and improve health care for the U.S. population, the viability and use of telemedicine in resource-constrained settings in rural and low- and middle-income populations must be further characterized.

It is acknowledged that the telemedicine specialty group from which the patient information was received is among the first of its kind in the United States and is unique in its scope, leading to a limitation of having a smaller than ideal sample size. However, it is believed that additional studies are expected to be performed after the COVID-19 pandemic. Regardless of sample size, this study will help to contrast the impact on clinical outcomes for T2DM patients by comparing those seen by telemedicine-trained and specialized physicians versus those who are not.

## Conclusion

The data imply that for telemedicine to be successful as a long-term medium of care, a precise standard of best practices needs to be implemented and documentation of objective data needs to be optimized and shareable. It is believed that successful long-term telemedicine care looks close to, if not identical, to the long-term care of any chronic illness, one where the patient has a relationship with his or her health care provider and is an active participant in the management of his or her own illness. If those are able to be established through the medium of telemedicine, it is hypothesized that there is a place for telemedicine in the long-term care of chronic illness.

The conclusion of this study is that telemedicine can be done well and that there is potential within telemedicine to disrupt the traditional model of care. However, even though these providers were uniquely trained in known telemedicine best practices, their patients still showed variable compliance and metabolic improvements. If uniquely trained providers have difficulties that hinder improvement in clinical outcomes with the current state of telemedicine, it cannot be expected for those without such training to have optimal outcomes.

If telemedicine is to become a mainstay in U.S. medicine, a standard of care needs to be designed and implemented, data need to be shareable and accessible, and providers who wish to continue to see patients via telemedicine should receive standardized fundamental training on telemedicine best practices.

Further study and implementation of well-defined best practices will elevate the overall standard of telemedicine for patients with T2DM and ensure their care is centered around self-empowerment to improve health and prevent disease exacerbations. It is hypothesized that diabetes specialty clinics that have the ability to reach far beyond the geographical constraints through telemedicine along with the proposed advanced training can provide patients with the greatest chance for success in their care plan.

## Authorship Contribution Statement

Conceptualization, methodology, and writing—original draft preparation, K.D.S., J.M.R., C.B.M., and B.B. Writing—editing, K.D.S., J.M.R., and C.B.M. Figures, K.D.S., J.M.R., and C.B.M. Supervision, B.B. All authors have read and agreed to the published version of the article.

## Abbreviations Used

BMI: body mass index
HbA1c: glycated hemoglobin
T2DM: type II diabetes mellitus
